# Characteristics of cytokines in the sciatic nerve stumps and DRGs after rat sciatic nerve crush injury

**DOI:** 10.1186/s40779-020-00286-0

**Published:** 2020-11-23

**Authors:** Rui-Rui Zhang, Sai-Ling Chen, Zhang-Chun Cheng, Yin-Ying Shen, Sheng Yi, Hui Xu

**Affiliations:** 1 Key Laboratory of Neuroregeneration of Jiangsu and Ministry of Education, Co-innovation Center of Neuroregeneration, Nantong University, Nantong, 226001 Jiangsu Province China; 2 College of Medicine, Nantong University, Nantong, 226001 Jiangsu Province China

**Keywords:** Peripheral nerve injury, Rat sciatic nerve crush injury, Sciatic nerve stumps, Dorsal root ganglia, Upstream cytokines

## Abstract

**Background:**

Cytokines are essential cellular modulators of various physiological and pathological activities, including peripheral nerve repair and regeneration. However, the molecular changes of these cellular mediators after peripheral nerve injury are still unclear. This study aimed to identify cytokines critical for the regenerative process of injured peripheral nerves.

**Methods:**

The sequencing data of the injured nerve stumps and the dorsal root ganglia (DRGs) of Sprague-Dawley (SD) rats subjected to sciatic nerve (SN) crush injury were analyzed to determine the expression patterns of genes coding for cytokines. PCR was used to validate the accuracy of the sequencing data.

**Results:**

A total of 46, 52, and 54 upstream cytokines were differentially expressed in the SNs at 1 day, 4 days, and 7 days after nerve injury. A total of 25, 28, and 34 upstream cytokines were differentially expressed in the DRGs at these time points. The expression patterns of some essential upstream cytokines are displayed in a heatmap and were validated by PCR. Bioinformatic analysis of these differentially expressed upstream cytokines after nerve injury demonstrated that inflammatory and immune responses were significantly involved.

**Conclusions:**

In summary, these findings provide an overview of the dynamic changes in cytokines in the SNs and DRGs at different time points after nerve crush injury in rats, elucidate the biological processes of differentially expressed cytokines, especially the important roles in inflammatory and immune responses after peripheral nerve injury, and thus might contribute to the identification of potential treatments for peripheral nerve repair and regeneration.

**Supplementary Information:**

The online version contains supplementary material available at 10.1186/s40779-020-00286-0.

## Background

Peripheral nerves are vulnerable tissues that are generally defenseless against traumatic injuries caused by bumping, stretching, crushing, and penetrating wounds as well as nontraumatic injuries caused by genetic, metabolic, infectious, and medically induced factors [1, 2]. Fortunately, unlike central nerves, peripheral nerves can regenerate and achieve certain functional recovery after injury, although full functional recovery is generally unexpected [3]. After peripheral nerve injury, distal nerve stumps undergo Wallerian degeneration. Activated Schwann cells and macrophages clear debris of axon and myelin sheaths. Axons of surviving neurons regrow toward target tissues for reinnervation [3, 4].

Cytokines are a broad category of immunomodulatory proteins or peptides, including chemokines, interferons, interleukins, lymphokines, and tumor necrosis factors. Cytokines play essential roles in inflammation and immune responses and participate in the regulation of the maturation, growth, and responsiveness of various cell populations [5, 6]. These molecules have been identified to be constitutively involved in the nervous system under various physiological and pathological conditions [7–10]. Cytokines are also critical for peripheral nerve injury and repair, as fine-tuned expression of cytokines modulates the cellular behaviors of Schwann cells, macrophages, and neurons and regulates debris clearance, axon growth, and peripheral nerve regeneration [11].

Understanding the molecular changes of these cellular mediators after peripheral nerve injury opens new possibilities to improve the repair of injured nerves and to minimize the induction of neuropathic pain [11]. To identify critical molecules that may be beneficial for peripheral nerve regeneration, we performed high-throughput analysis methods, such as RNA sequencing and microarray, to determine the gene changes after peripheral nerve injury [12–15]. These studies showed that many biological functions, such as cellular behavior, tissue/organ development, inflammation and immune responses, were significantly activated after nerve injury. Considering that cytokines are key molecules that regulate inflammation and immune responses, in the current study, previously obtained sequencing data of the injured nerve stumps of Sprague-Dawley (SD) rats subjected to sciatic nerve (SN) crush injury were analyzed to determine the expression patterns of genes coding for cytokines [13]. Moreover, considering that cytokines show retrograde transport to the neuronal bodies and affect neuronal activities, sequencing data of the dorsal root ganglia (DRGs) after rat SN crush injury were also jointly investigated [16]. Differentially expressed genes in the SNs and DRGs after nerve crush injury were identified, and upstream cytokines of these differentially expressed genes were determined by the Ingenuity Pathway Analysis (IPA) bioinformatic tool. Differentially expressed upstream cytokines at 1 day, 4 days, and 7 days after nerve crush injury were subjected to functional enrichment of Gene Ontology (GO) categories and Kyoto Enrichment of Genes and Genomes (KEGG) pathways according to the Database for Annotation, Visualization, and Integrated Discovery (DAVID).

## Materials and methods

### Sequencing data

RNA deep sequencing data of rat SNs at 0 h, 1 day, 4 days, 7 days, and 14 days after SN crush injury [13] were stored in the National Center for Biotechnology Information (NCBI) database with the accession number PRJNA394957 (SRP113121). Sequencing data of the rat DRGs at 0 h, 3 h, 9 h, 1 day, 4 days, and 7 days after SN crush injury [16] were stored in the NCBI database with the accession number PRJNA547681 (SRP200823). Differentially expressed genes in the SNs and DRGs at certain time points after nerve crush injury were selected by comparing their expression levels under the injured status with the expression levels under the uninjured status (0 h control). Genes with fold changes < 2 or > − 2 and an experimental false discovery rate (FDR) < 0.05 were defined as differentially expressed genes as previously demonstrated [13, 16].

### Bioinformatic analysis

Differentially expressed genes in the SNs and the DRGs were uploaded to the IPA bioinformatic tool (Ingenuity Systems, Inc., Redwood City, CA, USA) for core analysis. Upstream regulators of these differentially expressed genes were identified using Ingenuity Pathway Knowledge Base (IPKB)-based upstream regulator analysis. Upstream cytokines were then screened out. Genes coding for cytokines with fold changes < 2 or > − 2 at 1 day, 4 days, or 7 days compared with 0 h were defined as differentially expressed cytokines and were subjected to subsequent bioinformatic analyses.

Commonly differentially expressed cytokines in the SNs and the DRGs at 1 day, 4 days, or 7 days after SN crush injury were identified by the Venny 2.1.0 online bioinformatic tool (http://bioinfogp.cnb.csic.es/tools/venny/index.html) [17]. The expression profiles of these commonly differentially expressed cytokines were demonstrated by a heatmap. Signaling pathways and biological processes involved in differentially expressed upstream cytokines were identified by DAVID bioinformatic enrichment tools [18, 19].

### Animal surgery and collection of the DRGs and SN stumps

The conduction of rat SN crush injury and the collection of the SNs and the DRGs of the uninjured and injured rats were performed as previously described [13, 16]. A total of 24 adult male SD rats weighing 180–220 g were obtained from the Experimental Animal Center of Nantong University (Animal licenses no. SCXK [Su] 2014–0001 and SYXK [Su] 2012–0031) and subjected to animal surgery. Rats were randomly divided into 4 groups (0 h, 1 day, 4 days, and 7 days), with 6 rats in each group. Rats were anaesthetized intraperitoneally with a mixture of 85 mg/kg trichloroacetaldehyde monohydrate, 42 mg/kg magnesium sulfate, and 17 mg/kg sodium pentobarbital. SNs 10 mm above the bifurcation into the tibial and common fibular nerves were exposed by a skin incision in the left outer mid-thigh. Exposed SNs were crushed with forceps 3 times (a period of 10 s each time). At 1 day, 4 days, and 7 days after SN crush injury, the rats were sacrificed by decapitation. The rats in the 0-h group were subjected to sham surgery. The 6 rats in each group were divided into 3 replicates with 2 rats in each replicate for tissue collection. Rat SN segments of 5 mm in length at the crush sites as well as L4 to L6 DRGs were harvested for RNA isolation.

### RNA isolation and PCR validation

RNA was isolated from the rat SNs or l L4 to L6 DRG using Trizol reagent (Life Technologies, Carlsbad, CA, USA). Isolated RNA samples were reverse transcribed to cDNA using the Prime-Script reagent kit (TaKaRa, Dalian, Liaoning, China) and subjected to PCR experiments using an Applied Biosystems StepOne System (Applied Biosystems, Foster City, CA, USA) with SYBR Premix Ex *Taq* (TaKaRa) and specific primer pairs of target genes chemokine (C-X-C motif) ligand 10 (CXCL10) and interleukin 1 receptor antagonist (IL-1RN) and reference gene glyceraldehyde-3-phosphate dehydrogenase (GAPDH). The sequences of primer pairs were as follows: CXCL10, 5′-GAAGCACCATGAACCCAAGT-3′ (forward) and 5′-CAACATGCGGACAGGATAGA-3′ (reverse); IL-1RN, 5′-CTTACCTTCATCCGCTCCGA-3′ (forward) and 5′-GATCAGGCAGTTGGTGGTCAT-3′ (reverse); GAPDH 5′-ACAGCAACAGGGTGGTGGAC-3′ (forward) and 5′-TTTGAGGGTGCAGCGAACTT-3′ (reverse). The relative mRNA abundances of CXCL10 and IL-1RN were determined using the comparative 2^−ΔΔCt^ method, in which ΔCt = Ct_(injured)_-Ct_(uninjured)_ and ΔΔCt = Ct_(target gene)_-Ct_(reference gene)_ [20].

### Statistical analysis

Summarized PCR results are reported as the mean ± SEM with *n* = 3. Graphs were generated using GraphPad Prism 6.0 (GraphPad Software, Inc., San Diego, CA, USA). Kruskal-Wallis test was applied for statistical analysis, and *P* < 0.05 was considered statistically significant.

## Results

### Identification of differentially expressed upstream cytokines in the SNs and the DRGs following peripheral nerve injury

IPA bioinformatic analysis was applied to screen upstream cytokines of the differentially expressed genes in the SNs and the DRGs after nerve crush injury. The expression levels of genes coding for these upstream cytokines were further examined, and differentially expressed upstream cytokines in the SNs and the DRGs at 1 day, 4 days, and 7 days after nerve injury were identified (Supplementary Table 1).

**Fig. 1:**
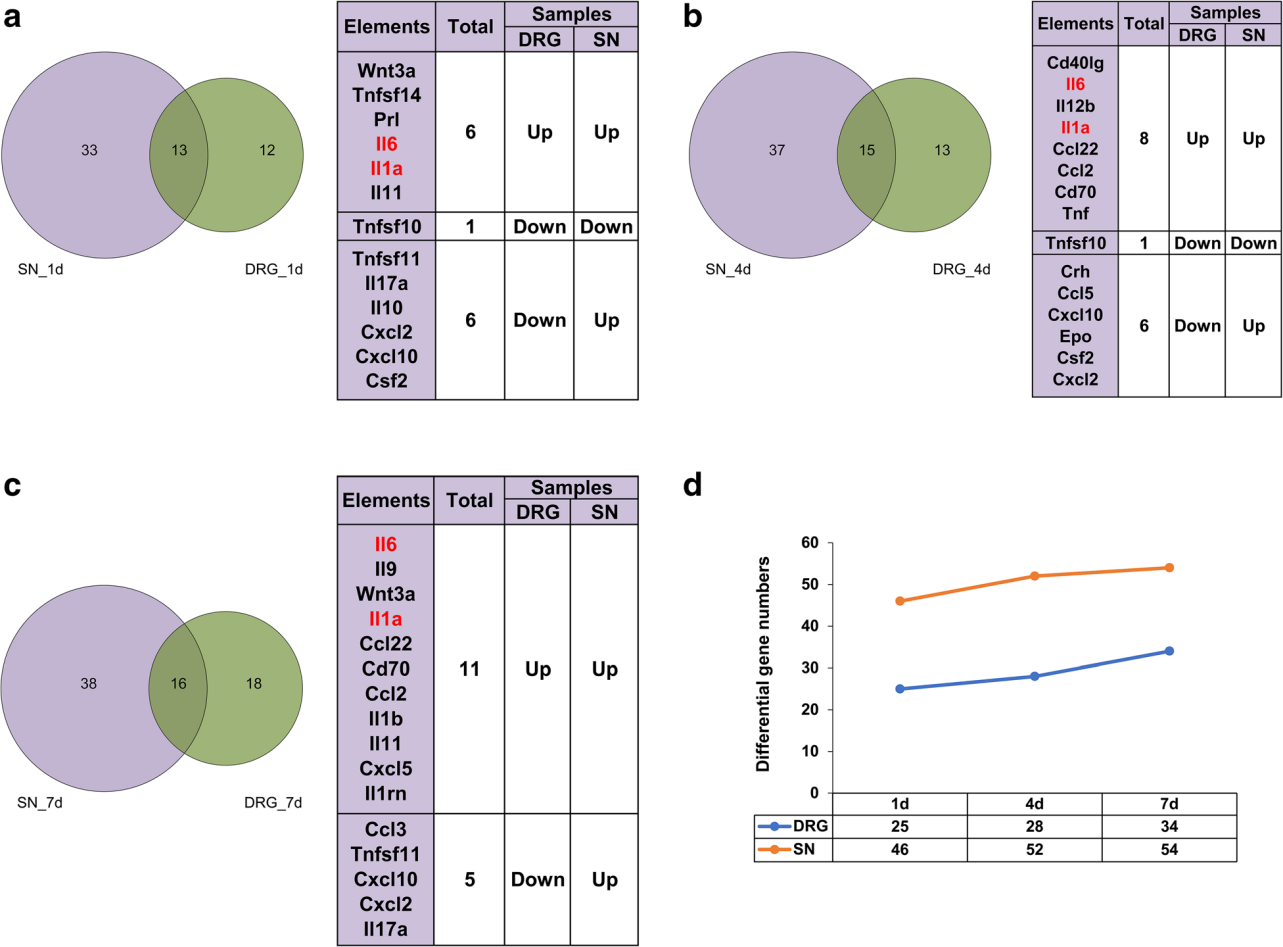
Overview of differentially expressed upstream cytokines in the SNs and DRGs after SN crush injury. Venn diagrams of differentially expressed upstream cytokines in the SNs and DRGs at (**a**) 1 day, (**b**) 4 days, and (**c**) 7 days after nerve injury. Overlapping cytokines in the SNs and DRGs are listed. Red color indicates upregulated genes at all tested time points. The numbers of differentially expressed upstream cytokines are listed (**d**). SN. Sciatic nerve; DRG. Dorsal root ganglia

Venn diagrams were generated to compare differentially expressed upstream cytokines in the SNs and the DRGs at certain time points after nerve injury and to obtain a comprehensive view of altered cytokines after rat SN crush injury (Fig. 1a-c). A total of 46 upstream cytokines were differentially expressed in the SNs at 1 day after nerve injury. At later time points, a relatively larger number of upstream cytokines were differentially expressed in the SNs (Fig. 1d). In the DRGs, a smaller group of upstream cytokines was differentially expressed compared with those in the SNs. The numbers of differentially expressed upstream cytokines also increased at later time points after nerve injury (Fig. 1d). Detailed investigation of these differentially expressed upstream cytokines showed that the majority of cytokines were upregulated and only a few cytokines were downregulated in the SNs. However, in the DRGs, the percentage of downregulated cytokines was much higher (Supplementary Table 1). The Venn diagram intersection identified cytokines that were differentially expressed in both the SNs and DRGs at the same time point. The expression changes of these SN and DRG intersecting cytokines are shown in addition to the Venn diagrams (Fig. 1a-c). Some cytokines, such as IL-6 and IL-1α, remained upregulated in the SNs and DRGs after nerve injury, while other cytokines, such as CXCL10, were upregulated in the SNs but downregulated in the DRGs (Fig. 1a-c).

### Demonstration of the expression patterns of upstream cytokines in the SNs and DRGs following peripheral nerve injury

**Fig. 2:**
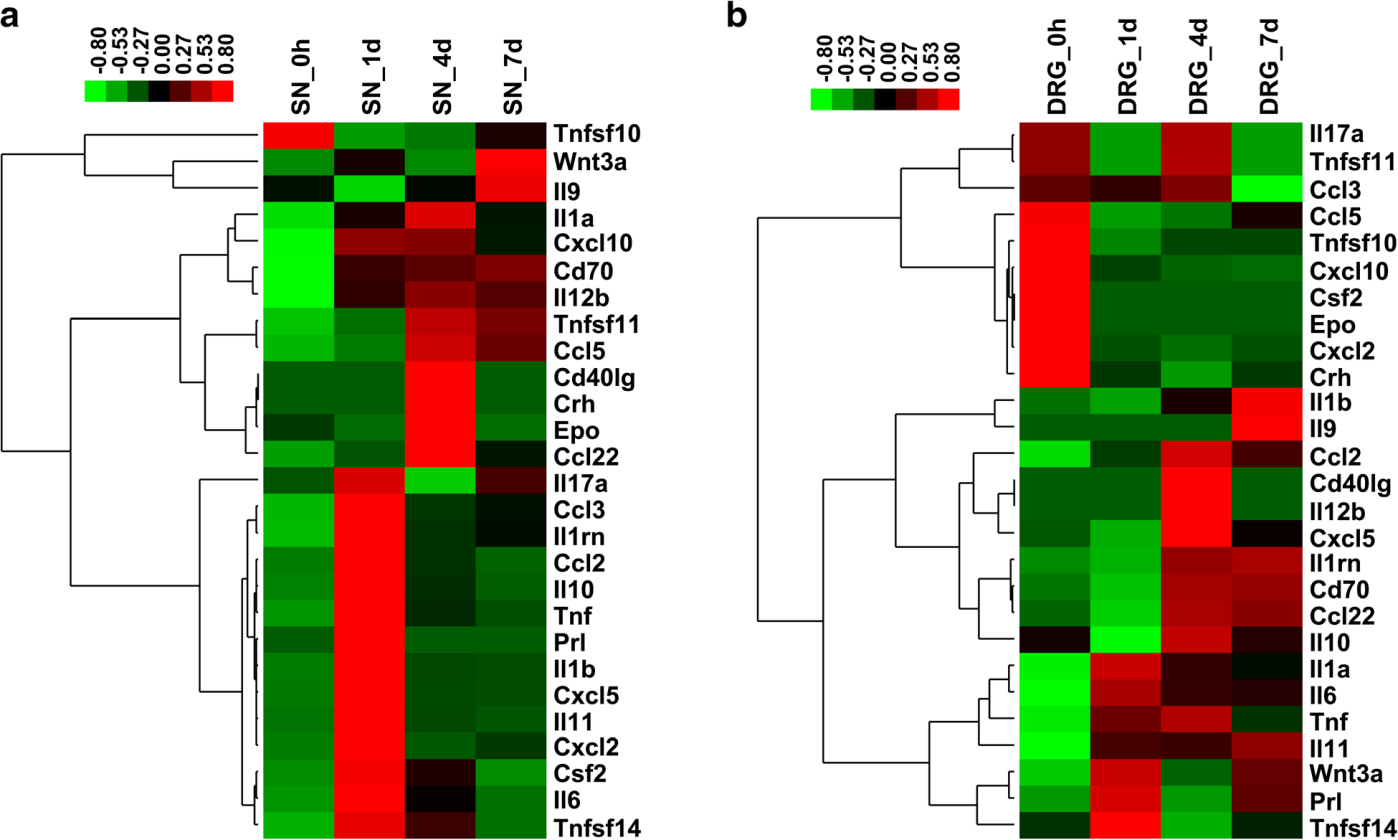
Heatmaps of the expression levels of commonly differentially expressed upstream cytokines in the SNs and DRGs. The relative expression levels of cytokines in (**a**) the SNs and (**b**) the DRGs at 0 h, 1 day, 4 days, and 7 days are displayed in colors. Green indicates downregulation, while red indicates upregulation. SN. Sciatic nerve; DRG. Dorsal root ganglia

To identify the dynamic changes in critical cytokines after peripheral nerve injury, we further studied the SN and DRG intersecting cytokines. A total of 27 cytokines were differentially expressed in both the SNs and DRGs at 1 day, 4 days, or 7 days after nerve injury. The expression levels of these cytokines were investigated and displayed in heatmaps (Fig. 2). Some cytokines showed similar expression trends in both the SNs and the DRGs. For example, tumor necrosis factor ligand superfamily member 10 (TNFSF10) was downregulated in both the SNs and the DRGs after nerve injury, CD40 ligand (CD40LG) was upregulated in both the SNs and the DRGs at 4 days after nerve injury, and IL-9 was upregulated in both the SNs and the DRGs at 7 days after nerve injury. Some cytokines, such as IL-1RN and C-C motif chemokine ligand 2 (CCL2), exhibited higher expression changes in the SNs than in the DRGs.

**Fig. 3:**
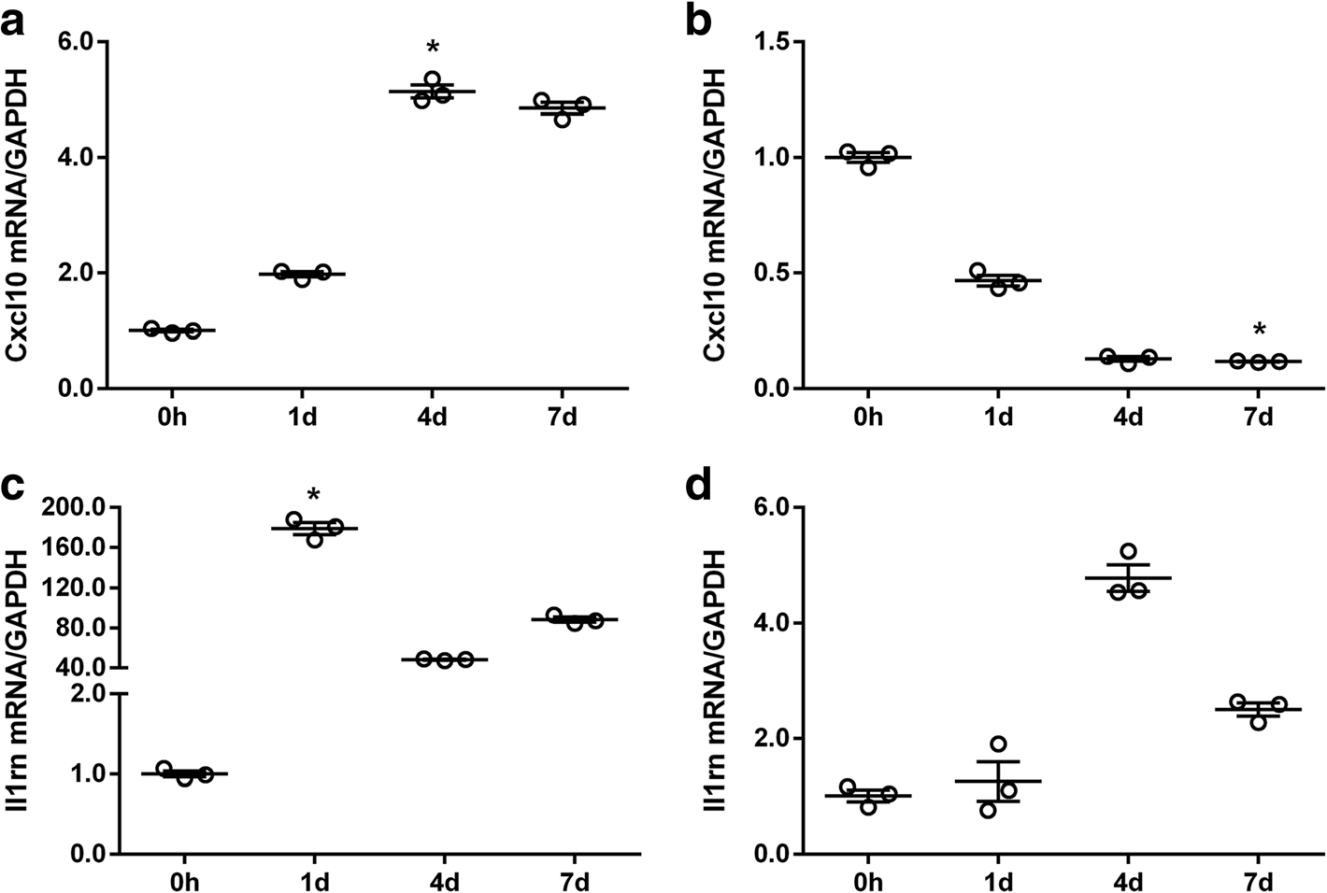
Validation of the expression levels of representative cytokines in the SNs and DRGs. The relative expression levels of CXCL10 in (**a**) the SNs and (**b**) the DRGs at 0 h, 1 day, 4 days, and 7 days after rat SN crush injury. The relative expression levels of IL-1RN in (**c**) the SNs and (**d**) the DRGs at 0 h, 1 day, 4 days, and 7 days after rat SN crush injury. The expression levels of CXCL10 and IL-1RN were normalized to that of GAPDH. Asterisks indicate significant differences (*P* < 0.05). Red lines indicate the expression trends revealed by sequencing. SN. Sciatic nerve; DRG. Dorsal root ganglia

The expression patterns of representative cytokines revealed by sequencing assays were further validated by quantitative PCR experiments. Independent SN crush injury experiments were performed in rats for the collection of the SNs and the DRGs and the conduction of PCR experiments. CXCL10, a cytokine whose mRNA expression was upregulated in the SNs but downregulated in the DRGs, and I11rn according to sequencing data, as well as IL-1RN, a cytokine whose mRNA expression was upregulated in both the SNs and the DRGs according to sequencing data, were selected for PCR validation. PCR experiments demonstrated that the mRNA levels of the cytokine CXCL10 were increased in the SNs (Fig. 3a) but decreased in the DRGs (Fig. 3b) following nerve injury. The relative abundances of genes coding for IL-1RN were upregulated in both the SNs (Fig. 3c) and the DRGs (Fig. 3d). These outcomes were consistent with the expression trends determined by sequencing data (shown in red lines), indicating that the sequencing data were highly accurate.

### Identification of significant signaling pathways of the differentially expressed upstream cytokines following peripheral nerve injury

**Fig. 4:**
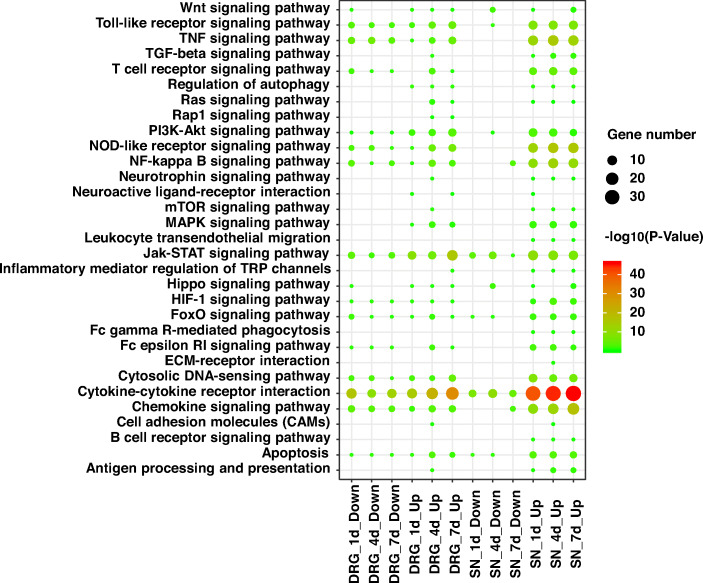
Activated nerve regeneration-related Kyoto Enrichment of Genes and Genomes (KEGG) signaling pathways of differentially expressed upstream cytokines in the SNs and DRGs. The sizes of the circles indicate the numbers of the differentially expressed upstream cytokines. Colors indicate the significance of the KEGG signaling pathways. SN. Sciatic nerve; DRG. Dorsal root ganglia

Bioinformatic analyses were performed to evaluate the significant signaling pathways of the differentially expressed upstream cytokines in the SNs and the DRGs after nerve injury. Activated signaling pathways that were related to nerve regeneration in the upregulated cytokines and the downregulated cytokines in the SNs and the DRGs were separately explored (Fig. 4). Cytokine-cytokine receptor interactions and chemokine signaling were the most strongly enriched signaling pathways. Other significantly enriched signaling pathways included Toll-like receptor signaling, TNF signaling, NOD-like receptor signaling, NF-κB signaling, and JAK-STAT signaling. These signaling pathways were most robustly involved in the upregulated upstream cytokines in the SNs.

### Identification of significant GO biological process categories and gene function regulatory networks of the differentially expressed upstream cytokines following peripheral nerve injury

**Fig. 5:**
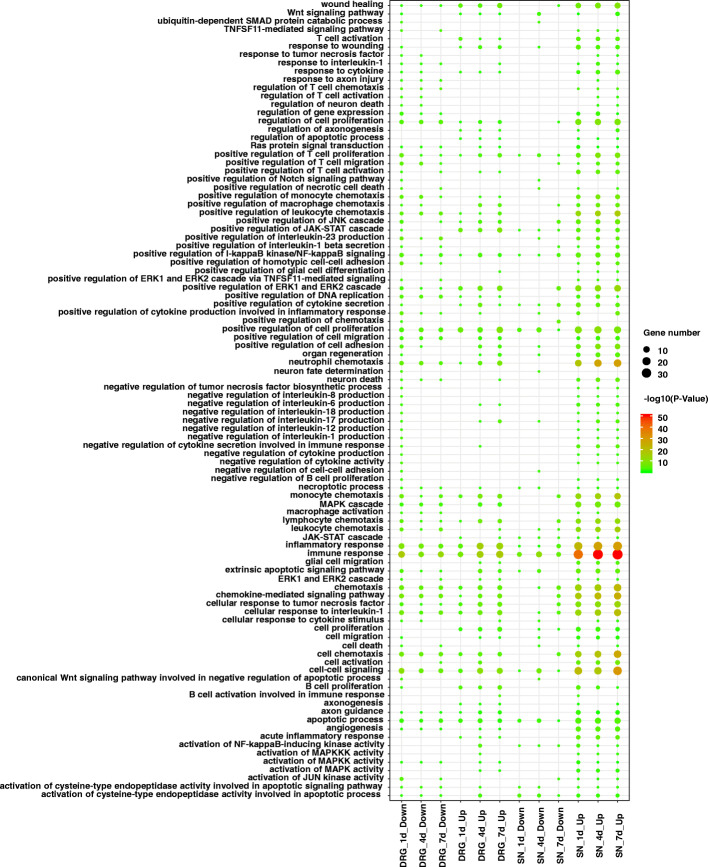
Activated nerve regeneration-related Gene Ontology (GO) biological process categories of differentially expressed upstream cytokines in the SNs and DRGs. The sizes of the circles indicate the numbers of differentially expressed upstream cytokines. Colors indicate the significance of GO biological process categories. SN. Sciatic nerve; DRG. Dorsal root ganglia

Critical nerve regeneration-related biological processes that occurred after SN crush injury were further discovered by categorizing differentially expressed upstream cytokines into GO terms. The inflammatory response and immune response were the most significant biological processes and were also most strongly involved in the upregulated upstream cytokines in the SNs (Fig. 5). Some other inflammatory response- and immune response-related biological processes, such as neutrophil chemotaxis, monocyte chemotaxis, and cellular response to IL-1, also exhibited low *P*-values, indicating the significance of inflammation and immune responses.

**Fig. 6:**
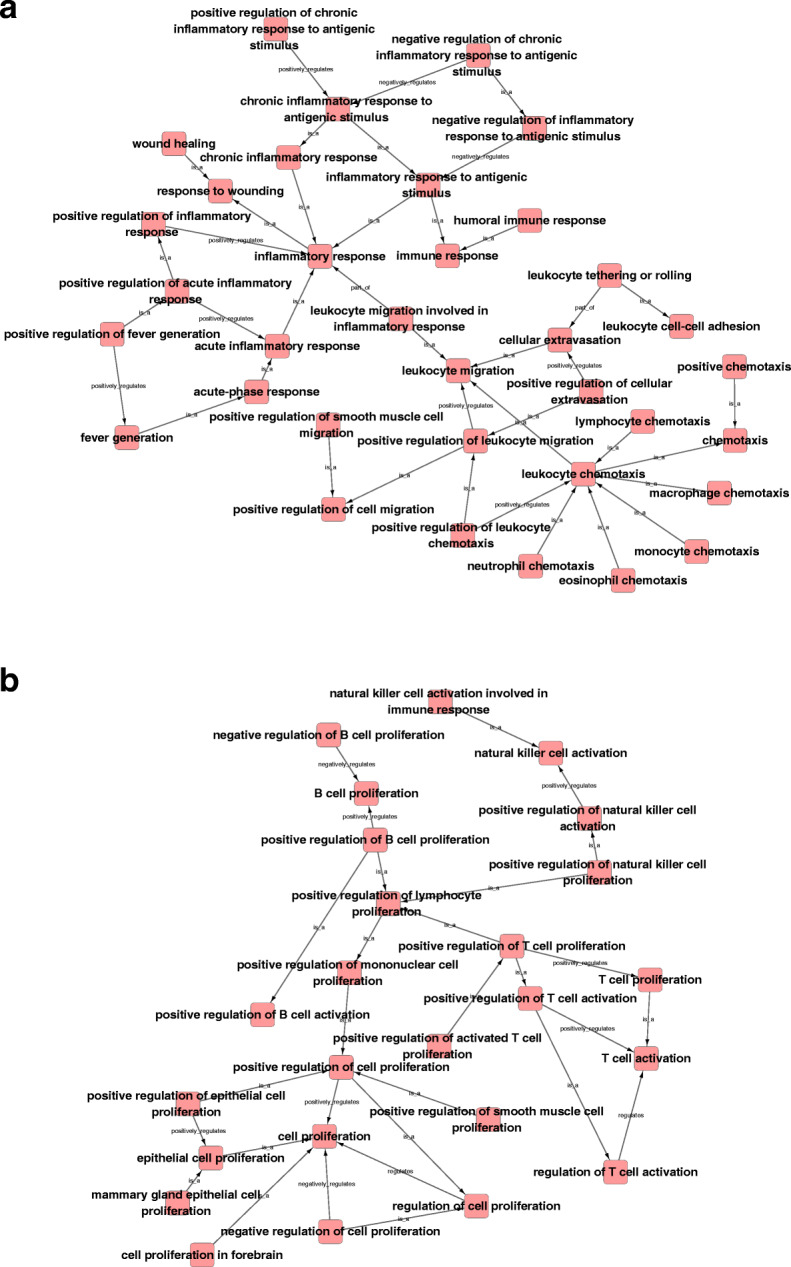
Gene function regulatory networks of significant Gene Ontology (GO) terms. The internal affiliation of (**a**) inflammatory response-related GO terms and (**b**) immune response-related GO terms

To further reveal the intrinsic links among gene functions, we performed a GO analysis on the differentially expressed cytokines in both the SNs and the DRGs at the same time point and constructed gene function regulatory networks (GO-Tree) for the significant GO terms (*P* < 0.05). The analysis showed that inflammation (Fig. 6a) and immune responses (Fig. 6b) were induced after peripheral nerve injury. The inflammation-centered network showed that both acute and chronic inflammatory responses were activated after nerve repair. The chemotaxis, migration, and extravasation of various types of cells, including lymphocytes, macrophages, and monocytes, contributed to activating the inflammatory response (Fig. 6a). The immune-centered network showed that many biological processes related to phenotype modulation of immune cells, such as the activation and proliferation of T cells, B cells, and natural killer cells, significantly participated in the generated network. This result indicated the critical roles of immune cells in nerve repair and regeneration (Fig. 6b).

## Discussion

Peripheral nerve injury induces the disconnection of axons from their cell bodies and leads to the disruption of axons and myelin sheaths in the injured nerve stumps as well as central chromatolysis and nuclear-associated changes in somas. With the rapid development of genomics and proteomics, the global genetic and molecular characteristics of a wide variety of physiological and pathological conditions, including peripheral nerve injury and regeneration, have been recognized. Moreover, some molecules that are critical for peripheral nerve repair have been identified by screening differentially expressed genes and/or proteins after nerve injury.

Differentially expressed cytokines in the injured SNs might promote the infiltration and polarization of monocytes, macrophages, and Schwann cells, enhance the clearance of axon and myelin debris, and promote axon regrowth and regeneration. Many cytokines were found to be upregulated in the injured nerve stumps. These cytokines might be secreted and released by Schwann cells and macrophages after peripheral nerve injury [21, 22]. These upregulated cytokines, including CCL2, leukemia inhibitory factor (LIF), tumor necrosis factor-α (TNF-α), IL-1α, IL-1β, and pancreatitis-associated protein III (Pap-III), promote the infiltration of monocytes and macrophages into injured nerve sites and contribute to the remodeling and reconstruction of the microenvironment surrounding the injured sites [21, 23–26]. In the current study, many other cytokines, including chemokine (C-C motif) ligand 12 (CCL12), C-X-C motif chemokine ligand 2 (CXCL2), and C-X-C motif chemokine ligand 3 (CXCL3), were found to be expressed at high levels in the injured nerve stumps after peripheral nerve injury, indicating the potential applications of these cytokines in treating peripheral nerve injury and promoting axon regrowth.

Moreover, it is worth noting that many cytokines might have opposing effects at multiple time points during peripheral nerve regeneration and represent a “double-edged sword” [11]. Our current study suggested that differentially expressed upstream cytokines in the injured SNs after peripheral nerve injury were highly related to inflammation and immune responses. Therefore, the controversial biological roles of cytokines might be due to the degree and timing of inflammation and immune responses induced by different expression levels of cytokines [11]. These results were consistent with our previous findings that robust immune and inflammatory responses were not only activated at the early stage after nerve injury but also remained activated over 14 days after nerve injury [27]. These outcomes indicated that to achieve orchestrated regulation of cytokines, it is important to obtain an overview of the expression patterns of cytokines in the injured nerve stumps at different time points after peripheral nerve injury.

In addition to affecting the injured nerve stumps and reconstructing the regenerative microenvironment, cytokines could influence the expression of neurotrophins and their receptors and thus could affect the neurite outgrowth of neurons [11]. For instance, the addition of IL-4 or interferon-γ (IFN-γ) to neurotrophin-4 (NT-4)-treated DRG neurons would increase NT-4-induced neurite outgrowth, and the addition of TNF-α to neurotrophin-treated DRG neurons would decrease neurotrophin-induced neurite outgrowth [28]. In addition, cytokine-induced inflammation and immune responses activate retrograde signaling and might induce the death or survival of DRG neurons [11, 29]. Consequently, in the current study, we also jointly determined the dynamic expression levels of cytokines in the DRGs and discovered some significantly changed cytokines, such as interferon alpha 4 (IFNA4), IL-6, and IL-24. Interestingly, some cytokines, such as CXCL10, were discovered to be upregulated in the nerve stumps but downregulated in the DRGs after nerve injury. CXCL10 could promote the invasion of lymphocytes and macrophages, affect myelination in a viral model of multiple sclerosis [30], and induce neuropathic pain in DRGs after chronic constriction injury [31]. Therefore, upregulated CXCL10 in the SNs after nerve injury may contribute to debris clearance in the injured nerve stumps, while downregulated CXCL10 in the DRGs might contribute to the reduction of neuropathic pain. Further functional studies would reveal the specific roles of these cytokines during peripheral nerve repair and regeneration and would provide new targets for the treatment of peripheral nerve injuries.

## Conclusions

In summary, the findings provided an overview of the dynamic changes in cytokines in the SNs and the DRGs at different time points after rat nerve crush injury, elucidated the biological processes of differentially expressed cytokines, especially the important roles in inflammatory and immune responses after peripheral nerve injury, and thus might contribute to the identification of potential treatments for peripheral nerve repair and regeneration.

## Supplementary Information

**Additional file 1:** **Supplementary Table 1.** List of differentially expressed upstream cytokines in the SNs and DRGs at 1 day, 4 days, and 7 days after rat SN crush injury.

## Data Availability

Sequencing data of the rat SNs and DRGs are available in the NCBI database with the accession numbers PRJNA394957 (SRP113121) and PRJNA547681 (SRP200823).

## Abbreviations

DRGs: Dorsal root ganglia
SN: Sciatic nerve
IPA: Ingenuity Pathway Analysis
GO: Gene Ontology
KEGG: Kyoto Enrichment of Genes and Genomes
DAVID: Database for Annotation, Visualization, and Integrated Discovery
FDR: False discovery rate
IPKB: Ingenuity pathway knowledge base
