# Supplementary material for: Characteristics of cytokines in the sciatic nerve stumps and DRGs after rat sciatic nerve crush injury
Source: Mil Med Res. 2020 Nov 23;7:57. doi: 10.1186/s40779-020-00286-0 (PMC7682062; doi:10.1186/s40779-020-00286-0)
Supplement: Supplementary file 1 — Additional file 1: Supplementary Table 1. List of differentially expressed upstream cytokines in the SNs and DRGs at 1 day, 4 days, and 7 days after rat SN crush injury. [file 40779_2020_286_MOESM1_ESM.docx]

**Supplementary Table 1.** List of differentially expressed upstream cytokines in the SNs and DRGs at 1 day, 4 days, and 7 days after rat SN crush injury

| 1 d | | | 4 d | | | 7 d | | |
| --- | --- | --- | --- | --- | --- | --- | --- | --- |
| Gene | Log ratio | FDR | Gene | Log ratio | FDR | Gene | Log ratio | FDR |
| SN | | | | | | | | |
| CCL12^a^ | 14.800 | 0.000 | CCL12^a^ | 12.409 | 0.000 | CCL12^a^ | 11.630 | 0.000 |
| CXCL2^a^ | 12.446 | 0.000 | CXCL5^a^ | 9.615 | 0.000 | CXCL2^a^ | 9.887 | 0.000 |
| CXCL3^a^ | 11.164 | 0.000 | CXCL2^a^ | 9.041 | 0.000 | XCL1^a^ | 9.833 | 0.000 |
| IL-10^a^ | 10.552 | 0.000 | CXCL3^a^ | 8.656 | 0.000 | CXCL5^a^ | 9.552 | 0.000 |
| TNFSF14^a^ | 9.445 | 0.000 | TNFSF14^a^ | 8.654 | 0.000 | CXCL3^a^ | 9.189 | 0.000 |
| IL-12B^a^ | 7.123 | 0.007 | IL-10^a^ | 8.317 | 0.000 | IL-17c^a^ | 8.328 | 0.001 |
| IL-1RN^a^ | 6.515 | 0.000 | IL-12B^a^ | 7.491 | 0.001 | IL-12B^a^ | 7.287 | 0.003 |
| CSF2^a^ | 6.509 | 0.243 | CD40LG^a^ | 7.118 | 0.020 | IL-10^a^ | 7.172 | 0.029 |
| CCL2^a^ | 6.353 | 0.000 | IFNG^a^ | 6.695 | 0.146 | TNFSF14^a^ | 6.772 | 0.059 |
| IL-1B^a^ | 6.263 | 0.000 | IL-1A^a^ | 6.565 | 0.000 | IL-2^a^ | 6.317 | 0.120 |
| IL-1A^a^ | 5.728 | 0.000 | CSF2^a^ | 5.361 | 0.531 | IFNG^a^ | 6.228 | 0.244 |
| EBI3^a^ | 5.203 | 0.000 | EBI3^a^ | 5.052 | 0.000 | IL-1A^a^ | 5.438 | 0.000 |
| IL-36B^a^ | 5.197 | 0.000 | IL-1RN^a^ | 4.870 | 0.000 | WNT3A^a^ | 5.218 | 0.029 |
| IFNβ1^a^ | 5.177 | 0.481 | OSM^a^ | 4.488 | 0.000 | IL-1RN^a^ | 5.205 | 0.000 |
| IFNA4^a^ | 5.139 | 0.480 | IL-36A^a^ | 4.383 | 0.530 | IL-17F^a^ | 4.911 | 0.241 |
| CXCL1^a^ | 5.127 | 0.000 | CCL2^a^ | 4.041 | 0.000 | WNT7A^a^ | 4.643 | 0.059 |
| IL-11^a^ | 4.997 | 0.000 | LIF^a^ | 3.971 | 0.000 | EBI3^a^ | 4.258 | 0.000 |
| IL-6^a^ | 4.961 | 0.000 | CRH^a^ | 3.921 | 0.531 | TNFSF11^a^ | 3.650 | 0.000 |
| OSM^a^ | 4.939 | 0.000 | TNFSF11^a^ | 3.906 | 0.000 | SLURP1^a^ | 3.650 | 0.003 |
| CCL3^a^ | 4.799 | 0.000 | IL-36B^a^ | 3.828 | 0.000 | CCL3^a^ | 3.494 | 0.000 |
| IL-2^a^ | 4.762 | 0.478 | IL-6^a^ | 3.658 | 0.000 | IL-1B^a^ | 3.375 | 0.000 |
| TNF^a^ | 4.377 | 0.000 | IL-1B^a^ | 3.496 | 0.000 | OSM^a^ | 3.306 | 0.000 |
| PRL^a^ | 4.305 | 0.479 | PF4^a^ | 3.458 | 0.000 | CD70^a^ | 2.775 | 0.061 |
| IL-17F^a^ | 3.940 | 0.478 | IL-18^a^ | 3.320 | 0.000 | CCL2^a^ | 2.620 | 0.000 |
| WNT3a^a^ | 3.926 | 0.242 | CCL22^a^ | 3.222 | 0.000 | LIF^a^ | 2.468 | 0.000 |
| LIF^a^ | 3.667 | 0.000 | CCL3^a^ | 3.158 | 0.000 | PF4^a^ | 2.463 | 0.000 |
| CCL6^a^ | 3.458 | 0.000 | CXCL10^a^ | 3.070 | 0.000 | CXCL10^a^ | 2.428 | 0.000 |
| PF4^a^ | 3.436 | 0.000 | FASLG^a^ | 2.658 | 0.000 | IL-36B^a^ | 2.360 | 0.030 |
| CXCL10^a^ | 3.109 | 0.000 | CD70^a^ | 2.658 | 0.081 | CCL4^a^ | 2.241 | 0.000 |
| IL-18^a^ | 3.093 | 0.000 | TNF^a^ | 2.643 | 0.000 | TNF^a^ | 2.109 | 0.000 |
| TIMP1^a^ | 3.087 | 0.000 | IL-11^a^ | 2.188 | 0.000 | CXCL14^a^ | 2.099 | 0.000 |
| CD70^a^ | 2.542 | 0.110 | CCL6^a^ | 2.173 | 0.000 | IL-18^a^ | 2.037 | 0.000 |
| LTA^a^ | 2.307 | 0.002 | CCL5^a^ | 2.073 | 0.000 | IL-6^a^ | 1.945 | 0.000 |
| TNFSF11^a^ | 2.027 | 0.012 | CXCL1^a^ | 2.052 | 0.000 | CCL22^a^ | 1.891 | 0.000 |
| IL-17A^a^ | 1.805 | 0.338 | EPO^a^ | 1.880 | 0.122 | CCL28^a^ | 1.775 | 0.152 |
| VAV3^a^ | 1.796 | 0.000 | TNFSF13^a^ | 1.874 | 0.000 | IL-11^a^ | 1.775 | 0.000 |
| CCL22^a^ | 1.307 | 0.000 | TIMP1^a^ | 1.663 | 0.000 | CCL5^a^ | 1.759 | 0.000 |
| TNFSF13^a^ | 1.221 | 0.000 | VAV3^a^ | 1.556 | 0.000 | FASLG^a^ | 1.422 | 0.000 |
| IL-33^a^ | 1.201 | 0.000 | TNFSF13b^a^ | 1.183 | 0.000 | CCL6^a^ | 1.412 | 0.000 |
| AIMP1^a^ | 1.111 | 0.000 | SPP1^a^ | 1.089 | 0.000 | TNFSF13^a^ | 1.282 | 0.000 |
| NAMPT^a^ | 1.063 | 0.000 | IL-7^a^ | 1.073 | 0.002 | VAV3^a^ | 1.204 | 0.000 |
| IL-21^b^ | -1.121 | 0.014 | WNT4^b^ | -1.223 | 0.022 | IL-17A^a^ | 1.191 | 0.570 |
| CNTF^b^ | -1.289 | 0.000 | TNFSF10^b^ | -1.490 | 0.000 | SCGB1A1^a^ | 1.191 | 0.213 |
| TNFSF10^b^ | -1.749 | 0.000 | CTF1^b^ | -1.844 | 0.000 | IL-9^a^ | 1.191 | 0.570 |
| TNFSF15^b^ | -2.474 | 0.000 | IL-16^b^ | -2.032 | 0.000 | DKK3^a^ | 1.183 | 0.000 |
| IL-9^b^ | -4.936 | 0.622 | TNFSF15^b^ | -2.097 | 0.000 | CXCL1^a^ | 1.170 | 0.000 |
|  |  |  | WNT1^b^ | -2.249 | 0.040 | FAM3B^a^ | 1.039 | 0.236 |
|  |  |  | IL-21^b^ | -2.590 | 0.000 | TIMP1^a^ | 1.013 | 0.000 |
|  |  |  | CNTF^b^ | -2.722 | 0.000 | MIF^b^ | -1.046 | 0.000 |
|  |  |  | IL-12A^b^ | -2.802 | 0.000 | IL-16^b^ | -1.171 | 0.000 |
|  |  |  | IL-17A^b^ | -3.913 | 0.574 | TNFSF15^b^ | -1.748 | 0.000 |
|  |  |  | CSF3^b^ | -5.724 | 0.095 | CNTF^b^ | -2.480 | 0.000 |
|  |  |  |  |  |  | CCL19^b^ | -2.482 | 0.000 |
| **DRG** | | | | | | | | |
| CCL1^a^ | 7.672 | 0.052 | IL-5^a^ | 8.584 | 0.005 | PRLH^a^ | 8.197 | 0.090 |
| IFNA2^a^ | 6.993 | 0.097 | PRLH^a^ | 8.247 | 0.074 | IL-24^a^ | 7.583 | 0.000 |
| IL-6^a^ | 6.832 | 0.000 | CD40LG^a^ | 7.196 | 0.019 | IFNA4^a^ | 7.019 | 0.091 |
| PRL^a^ | 5.767 | 0.178 | IL-22^a^ | 7.147 | 0.075 | IL-5^a^ | 6.534 | 0.308 |
| IL-24^a^ | 4.839 | 0.000 | IFNA4^a^ | 7.069 | 0.075 | IL-6^a^ | 6.316 | 0.000 |
| WNT3a^a^ | 4.803 | 0.097 | IL-24^a^ | 6.951 | 0.000 | IFNβ1^a^ | 6.058 | 0.311 |
| IL-1a^a^ | 3.991 | 0.000 | IL-6^a^ | 6.395 | 0.000 | IL-9^a^ | 6.037 | 0.311 |
| CTF2^a^ | 2.669 | 0.103 | IL-12B^a^ | 4.247 | 0.524 | PRL^a^ | 5.185 | 0.307 |
| CXCL14^a^ | 1.256 | 0.000 | IL-1a^a^ | 3.430 | 0.000 | IL-22^a^ | 5.097 | 0.559 |
| IFNK^a^ | 1.183 | 0.021 | WNT3a^a^ | 2.857 | 0.524 | CSF3^a^ | 4.824 | 0.311 |
| TNFSF14^a^ | 1.084 | 0.509 | CCL22^a^ | 1.800 | 0.000 | WNT3a^a^ | 4.391 | 0.169 |
| IL-11^a^ | 1.010 | 0.121 | CCL2^a^ | 1.557 | 0.000 | IL-1a^a^ | 3.123 | 0.000 |
| IL-12A^b^ | -1.086 | 0.314 | CSF1^a^ | 1.481 | 0.000 | IL-12A^a^ | 1.775 | 0.001 |
| TNFSF10^b^ | -1.385 | 0.000 | CXCL14^a^ | 1.479 | 0.000 | IL-36RN^a^ | 1.672 | 0.220 |
| IL-17b^b^ | -1.501 | 0.305 | CD70^a^ | 1.459 | 0.295 | CCL22^a^ | 1.672 | 0.001 |
| CCL19^b^ | -1.501 | 0.003 | CCL11^a^ | 1.450 | 0.000 | CSF1^a^ | 1.528 | 0.000 |
| CCL5^b^ | -2.153 | 0.000 | SLURP1^a^ | 1.275 | 0.029 | CD70^a^ | 1.409 | 0.333 |
| CXCL10^b^ | -2.365 | 0.000 | LTA^a^ | 1.137 | 0.450 | CCL2^a^ | 1.202 | 0.000 |
| TNFSF11^b^ | -2.376 | 0.036 | IL-17C^a^ | 1.137 | 0.450 | IL-1B^a^ | 1.198 | 0.000 |
| CXCL2^b^ | -3.724 | 0.002 | TNF^a^ | 1.000 | 0.002 | IL-11^a^ | 1.158 | 0.060 |
| WNT1^b^ | -3.882 | 0.369 | TNFSF10^b^ | -1.060 | 0.000 | CXCL5^a^ | 1.087 | 0.493 |
| EPO^b^ | -4.641 | 0.371 | CRH^b^ | -1.267 | 0.000 | CTF2^a^ | 1.087 | 0.654 |
| IL-17A^b^ | -4.927 | 0.371 | CCL5^b^ | -1.737 | 0.001 | CCL11^a^ | 1.065 | 0.001 |
| IL-10^b^ | -5.673 | 0.368 | CXCL10^b^ | -3.079 | 0.000 | IFNK^a^ | 1.035 | 0.049 |
| CSF2^b^ | -6.302 | 0.372 | EPO^b^ | -4.641 | 0.356 | IL-1RN^a^ | 1.010 | 0.000 |
|  |  |  | CSF2^b^ | -6.302 | 0.357 | TNFSF10^b^ | -1.059 | 0.000 |
|  |  |  | IL-17b^b^ | -7.245 | 0.035 | IL-21^b^ | -1.291 | 0.134 |
|  |  |  | CXCL2^b^ | -7.790 | 0.000 | CCL3^b^ | -1.372 | 0.033 |
|  |  |  |  |  |  | TNFSF11^b^ | -2.372 | 0.034 |
|  |  |  |  |  |  | CXCL10^b^ | -3.337 | 0.000 |
|  |  |  |  |  |  | CXCL2^b^ | -3.720 | 0.002 |
|  |  |  |  |  |  | EPO^b^ | -4.641 | 0.357 |
|  |  |  |  |  |  | IL-17A^b^ | -4.927 | 0.357 |
|  |  |  |  |  |  | CSF2^b^ | -6.302 | 0.358 |

FDR: False discovery rate; SN: Sciatic nerve; DRG: Dorsal root ganglia. ^a^ Up-regulated upstream cytokines; ^b^ Down-regulated upstream cytokines.
